# Supplementary material for: Rad9/53BP1 promotes DNA repair via crossover recombination by limiting the Sgs1 and Mph1 helicases
Source: Nat Commun. 2020 Jun 23;11:3181. doi: 10.1038/s41467-020-16997-w (PMC7311424; doi:10.1038/s41467-020-16997-w)
Supplement: Supplementary file 1 — Supplementary Information [file 41467_2020_16997_MOESM1_ESM.pdf]

Supplementary Information to:

Rad9/53BP1 promotes DNA repair via crossover recombination by limiting the Sgs1 and Mph1 helicases.

Ferrari et al

CONTENTS

Supplementary Tables 1-6

Supplementary Figs. 1-5

Supplementary Fig. Legends

Supp. Table 1: Summary of DSB-induced recombination events in asynchronous cells (related to Fig. 1b, c)

			**Diploid 1**		**Diploid 2**		**Diploid 3**		**Diploid 4**	
			wild type	*rad9Δ*	*wild type*	*rad9Δ*	*wild type*	*rad9Δ*	*wild type*	*rad9Δ*
**White/Red sectored colonies**	**BIR**	Hph^SNat^R/Hph^RNat^R	1	0	2	0	2	1	3	3
		Hph^RNat^R/Hph^SNat^R	3	3	3	4	4	4	4	0
		Hph^SNat^R/Hph^SNat^R	0	0	0	0	0	0	0	0
		**Total no. of BIR**	**4**	**3**	**5**	**4**	**6**	**5**	**7**	**3**
	**BIR - like**	Hph^RNat^S/Hph^RNat^R	1	0	3	0	4	4	5	1
		Hph^RNat^R/Hph^RNat^S	0	0	1	0	1	0	0	0
		Hph^RNat^S/Hph^RNat^S	0	0	0	0	0	0	0	0
		**Total no. of BIR - like**	**1**	**0**	**4**	**0**	**5**	**4**	**5**	**1**
	**CO**	Hph^SNat^R/Hph^RNat^S	10	4	9	5	6	4	4	2
		Hph^RNat^S/Hph^SNat^R	2	0	3	3	0	0	2	0
		**Total no. of CO**	**12**	**4**	**12**	**8**	**6**	**4**	**6**	**2**
	**Total no. of NCO**		**34**	**16**	**62**	**9**	**41**	**16**	**52**	**14**
	**Chromosome loss** (Met^- or Trp^-)		0	0	0	0	0	0	0	0
	**White/ red colonies analyzed**		**51**	**23**	**83**	**21**	**58**	**29**	**70**	**20**
**Red colonies - recombinants**	**BIR**	Hph^SNat^R/Hph^RNat^R	0	0	3	1	3	2	4	1
	**BIR - like**	Hph^RNat^S/Hph^RNat^R	5	0	0	0	1	0	0	0
	**CO**	Hph^SNat^R/Hph^RNat^S	14	4	12	4	18	8	18	10
	**Total no. of NCO**		98	33	58	29	78	27	64	32
	**Chromosome loss** (Met^- or Trp^-)		0	0	0	0	0	0	0	0
	Unclassified LOH		1	13	3	8	5	4	9	2
	**Red colonies analyzed**		**118**	**50**	**76**	**42**	**105**	**41**	**95**	**45**
**White colonies**	**BIR**	Hph^SNat^R/Hph^RNat^R	0	0	0	0	0	1	2	0
	**BIR - like**	Hph^RNat^S/Hph^RNat^R	0	1	0	0	0	0	1	0
	**CO**	Hph^SNat^R/Hph^RNat^S	6	2	10	0	10	2	12	0
	**Total no. of NCO**		88	170	67	150	88	172	81	160
	**Chromosome loss** (Met^- or Trp^-)		0	0	0	0	0	0	0	0
	Unclassified LOH		13	8	10	11	7	6	7	5
	**White colonies analyzed**		**107**	**181**	**87**	**161**	**105**	**181**	**103**	**165**
Red (Not recombinant)			12	5	7	5	5	4	1	4
Total colonies			288	259	253	229	273	255	269	234
**Total recombinants**			**276**	**254**	**246**	**224**	**268**	**251**	**268**	**230**

Supp. Table 2: Distribution of different colony types following 1.5 h of DSB induction in asynchronous cells (related to Fig. 1b, c)

Strain		Recombinant			Total no. of recombinants	Non-recombinants	Total no. of colonies	Plating efficiency
		White	Red	Red/white sectored				
wild - type	Diploid 1	107 (37.1%)	118 (40.9%)	51 (17.7%)	276 (95.8%)	12 (4.2%)	288	82.6%
	Diploid 2	87 (34.4%)	76 (30%)	83 (32.8%)	246 (97.2%)	7 (2.8%)	253	83%
	Diploid 3	105 (38.5%)	105 (38.5%)	58 (21.2%)	268 (98.1%)	5 (1.8%)	273	87%
	Diploid 4	103 (38.2%)	95 (35.3%)	70 (26%)	268 (99.6%)	1 (0.4%)	269	86.2%
rad9Δ	Diploid 1	181 (69.9%)	50 (19.3%)	23 (8.9%)	254 (98.1%)	5 (1.9%)	259	74.6%
	Diploid 2	161 (70.3%)	42 (18.3%)	21 (9.2%)	224 (97.8%)	5 (2.2%)	229	74.4%
	Diploid 3	181 (70.9%)	41 (16.1%)	29 (11.4%)	251 (98.4%)	4 (1.6%)	255	75%
	Diploid 4	165 (70.5%)	45 (19.2%)	20 (85.4%)	230 (98.3%)	4 (1.7%)	234	73.1%

Supp. Table 3: Summary of DSB-induced recombination events in nocodazole arrested cells (related to Fig. 1b, c)

			Diploid 1		Diploid 2	
			wild type	*rad9Δ*	*wild type*	*rad9Δ*
White/Red sectored colonies	BIR	$Hph^{S}Nat^{R}/Hph^{R}Nat^{R}$	2	0	1	0
White/Red sectored colonies	BIR	$Hph^{R}Nat^{R}/Hph^{S}Nat^{R}$	3	2	3	3
White/Red sectored colonies	BIR	$Hph^{S}Nat^{R}/Hph^{S}Nat^{R}$	0	0	0	0
White/Red sectored colonies	BIR	**Total no. of BIR**	**5**	**2**	**4**	**3**
White/Red sectored colonies	BIR - like	$Hph^{R}Nat^{S}/Hph^{R}Nat^{R}$	3	0	1	0
White/Red sectored colonies	BIR - like	$Hph^{R}Nat^{R}/Hph^{R}Nat^{S}$	0	0	0	0
White/Red sectored colonies	BIR - like	$Hph^{R}Nat^{S}/Hph^{R}Nat^{S}$	0	0	0	0
White/Red sectored colonies	BIR - like	**Total no. of BIR - like**	**3**	**0**	**1**	**0**
White/Red sectored colonies	CO	$Hph^{S}Nat^{R}/Hph^{R}Nat^{S}$	2	2	1	0
White/Red sectored colonies	CO	$Hph^{R}Nat^{S}/Hph^{S}Nat^{R}$	0	1	2	2
White/Red sectored colonies	CO	**Total no. of CO**	**2**	**3**	**3**	**2**
White/Red sectored colonies	**Total no. of NCO**		**47**	**18**	**45**	**13**
White/Red sectored colonies	**Chromosome loss** (Met^{-} or Trp^{-})		0	0	0	0
White/Red sectored colonies	**White/ red colonies analyzed**		**57**	**23**	**53**	**18**
Red colonies - recombinants	BIR	$Hph^{S}Nat^{R}/Hph^{R}Nat^{R}$	3	1	3	0
Red colonies - recombinants	BIR - like	$Hph^{R}Nat^{S}/Hph^{R}Nat^{R}$	1	0	1	0
Red colonies - recombinants	CO	$Hph^{S}Nat^{R}/Hph^{R}Nat^{S}$	10	2	15	2
Red colonies - recombinants	**Total no. of NCO**		79	21	83	36
Red colonies - recombinants	**Chromosome loss** (Met^{-} or Trp^{-})		0	0	0	0
Red colonies - recombinants	Unclassified LOH		10	11	15	3
Red colonies - recombinants	**Red colonies analyzed**		**103**	**35**	**117**	**41**
White colonies	BIR	$Hph^{S}Nat^{R}/Hph^{R}Nat^{R}$	1	1	2	0
White colonies	BIR - like	$Hph^{R}Nat^{S}/Hph^{R}Nat^{R}$	0	0	0	0
White colonies	CO	$Hph^{S}Nat^{R}/Hph^{R}Nat^{S}$	4	0	5	0
White colonies	**Total no. of NCO**		97	163	96	180
White colonies	**Chromosome loss** (Met^{-} or Trp^{-})		0	0	0	0
White colonies	Unclassified LOH		5	6	7	2
White colonies	**White colonies analysed**		**107**	**170**	**110**	**182**
Red (Not recombinant)			9	8	12	6
Total colonies			276	236	292	247
**Total recombinants**			**267**	**228**	**280**	**241**

Supp. Table 4: Distribution of different colony types following 1.5 h of DSB induction in nocodazole arrested cells (related to Fig. 1b, c)

Strain		Recombinant			Total no. of recombinants	Non-recombinants	Total no. of colonies	Plating efficiency
		White	Red	Red/white sectored				
wild - type	Diploid 1	107 (38.8%)	103 (37.3%)	57 (20.7%)	267 (96.7%)	9 (3.3%)	276	86,5%
	Diploid 2	110 (37.7%)	117 (40.1%)	53 (18.1%)	280 (95.9%)	12 (4.1%)	292	85,5%
rad9Δ	Diploid 1	170 (72%)	35 (14.8%)	23 (9.7%)	228 (96.6%)	8 (3.4%)	236	74,6%
	Diploid 2	182 (73.7%)	41 (16.6%)	18 (7.3%)	241 (97.6%)	6 (2.4%)	247	74%

Supplementary Table 5. List of yeast strains described in this work.

Strain name	Parental strain	Genotype	Source
Y1600	JKM179	*MATa ho hml::ADE1 hmr::ADE1 ade1-100 leu2-3, 112 lys5, trp1::hisG ura3-52 lys5::ade3::GAL10::HO*	Lee et al., 1998
Y603	tGI354	*ho hml::ADE1 MAT***a***-inc hmr::ADE1 ade1 leu2-3,112 lys5 trp1::hisG ura3-52 ade3::GAL10::HO (arg5,6::MAT***a***::HPH)*	Ira et al., 2003
Y3172	JRL092	*ho mat::hisG hml::hisG HMRa-stk ura3Δ851 trp1Δ63 leu2Δ::KAN ade3::GAL10::HO can1,1-1446::HOcs::HPH ykl215c::LEU2::can1Δ289*	Lydeard et al., 2007
Y3480	JRL092	*ho mat::hisG hml::hisG HMRa-stk ura3Δ851 trp1Δ63 leu2Δ::NAT ade3::GAL10::HO can1,1-1446::HOcs::HPH ykl215c::LEU2::can1Δ289*	This study
Y4197	Y3480	*rad9::TRP1*	This study
Y3513	Y3480	*rad53::K227A::KANMX6@RAD53 chk1::TRP1*	This study
Y3308	Y3172	*pif1::TRP1*	This study
Y3387	Y3172	*sgs1::NAT*	This study
Y3475	Y3172	*mph1::NAT*	This study
Y3981	Y3172	*mph1::NAT sgs1::URA3*	This study
Y3454	Y3172	*sgs1::NAT rad9::TRP1*	This study
Y3488	Y3172	*mph1::NAT rad9::TRP1*	This study
Y3982	Y3172	*sgs1::URA3 mph1::NAT rad9::TRP1*	This study
Y4377	Y3480	*sgs1-F1192D rad9:: TRP1 mph1::KANMX6*	This study
Y4351	Y3480	*sgs1-F1192D rad9::TRP1*	This study
Y4314	Y3480	*sgs1-F1192D*	This study
Y4302	Y3172	*rad9::NAT rad9-3xHA::TRP1*	This study
Y4312	Y3172	*rad9::NAT rad9-2Ala-3xHA::TRP1*	This study
Y4304	Y3172	*rad9::NAT rad9-7xA-3xHA::TRP1*	This study
Y4251	Y4197	*rad9::TRP1 SGS1-13xMYC::KANMX6*	This study
Y4249	Y3480	*SGS1-13xMYC::KANMX6*	This study
Y4648	Y3480	*MPH1-13xMYC::KANMX6*	This study
Y4649	Y4197	*rad9::TRP1 MPH1-13xMYC::KANMX6*	This study
Y4279	Y1600	*MATa RAD9::HPH SGS1-13xMYC::KANMX6*	This study
Y4046	Y1600	*MATa SGS1::13xMYC::KANMX6*	This study
Y4283	Y1600	*MATa RPA1::13xMYC::KANMX6*	This study
Y4289	Y1600	*MATa RPA1::13xMYC::KANMX6 RAD9::HPH*	This study
Y3892	Y1600	*MATa RAD52::3xHA::KANMX6*	This study
Y3890	Y1600	*MATa RAD52::3xHA::KANMX6 rad9::HPH*	This study
Y4332	Y1600	*MATa sgs1-F1192D*	This study
Y4488	Y1600	*MATa sgs1-F1192D rad9::HPH*	This study
Y2995	Y603	*rad9::NAT*	This study
Y4376	Y603	*sgs1::KANMX6 mph1::TRP1*	This study
Y4429	Y603	*rad9::NAT sgs1::KANMX6 mph1::TRP1*	This study
Y3412	LSY2205-11C	*MAT alpha, ade2-I lys2::GAL-I-SCEI his3::HPHMX4*	Ho et al. 2010
Y3414	LSY2543	*MAT a, met22::KlTRP1 ade2-n his3::NATMX4*	Ho et al. 2010
Y3831	Y3412	*rad9::KANMX6*	This study
Y3849	Y3414	*rad9::KANMX6*	This study

Supplementary Table 6.

List of the oligonucleotides used for ChIP and DSB resection analyses in JKM strain

Name	Sequence (5′-3′)	Distance from DSB	Source
QMAT1F	CCTGGTTTTGGTTTTGTAGAGTGG	0.15 kb (Chr III)	Kim et al. 2007
QMAT1R	GAGCAAGACGATGGGGAGTTTC	0.15 kb (Chr III)	Kim et al. 2007
QMAT1.4F	ATTCCGTAAAGTCATAAGCACCCAC	1.4 kb (Chr III)	This study
QMAT1.4R	GTCCGCAGCTTGATTGAAAATGTTG	1.4 kb (Chr III)	This study
QMAT2.F	ATTGCGACAAGGCTTCACCC	4.8 kb (Chr III)	Kim et al. 2007
QMAT2.R	CCACATCACAGGTTTATTGGTTCC	4.8 kb (Chr III)	Kim et al. 2007
QMAT3.1.F	GGTTCAACAACTGCCACCTTTG	10 kb (Chr III)	Dibitetto et al. 2016
QMAT3.1.R	GGTGATGGAGATGGAGTAGGAACG	10 kb (Chr III)	Dibitetto et al. 2016
QMAT4F	CGTCTTCTCAGCGAACAACAGC	15 kb (Chr III)	Kim et al. 2007
QMAT4R	GCAATAACCCACGGAAACACTG	15 kb (Chr III)	Kim et al. 2007
QKCC4F	TCGTATCAGGTCTGCCCTATGAA	Chr III	This study
QKCC4R	CTCTGGAAATTTCGGTGTCATTG	Chr III	This study
QCan1F (P8)	CAATGGTGTTAGCTTTGCTGCC	Chr XI (JRL92 strain)	This study
QCan1R (P9)	TTGCCTCAATGTCTCTTCTATCGG	Chr XI (JRL92 strain)	This study
P3	GACATGGAGGCCCAGAATAC	Chr V (JRL92 strain)	This study
P4	GAGCCGTAATTTTTGCTTCG	Chr V (JRL92 strain)	This study
QMAT CE R	CCACTCTACAAAACCAAAACCAGG	Chr V (JRL92 strain)	This study
Q MAT JRL092 CE F1	GTATTCTGGGCCTCCATGTC	Chr V (JRL92 strain)	This study

List of the oligonucleotides used for the primer extension assay in the JRL092 strains.

Name	Sequence (5′-3′)	Position	Source
P1	GAGGATACGTTCTCTATGGAG	*CAN1* on Chr V	This study
P2	GTCTTTGGTTCATGATCTTCCC	*CAN1* on Chr XI	This study
TLC1For	CTACTTCTCCTAATGCCTTCG	*TLC1* on Chr II	This study
TLC1Rev	TGGTCCTATCAGTAACACTCG	*TLC1* on Chr II	This study

List of oligonucleotides used for CO/NCO analysis.

Name	Sequence (5′-3′)	Position	Source
P5	CAAGACTGTCAAGGAGGGTATTC	Chr V	This study
P6	TCAGGGATACCAGCATACTC	Chr V	This study
P7	GATGCCCTTGTTTTGTTTACTG	Chr III	This study

Ferrari et al SUP. Fig. 1

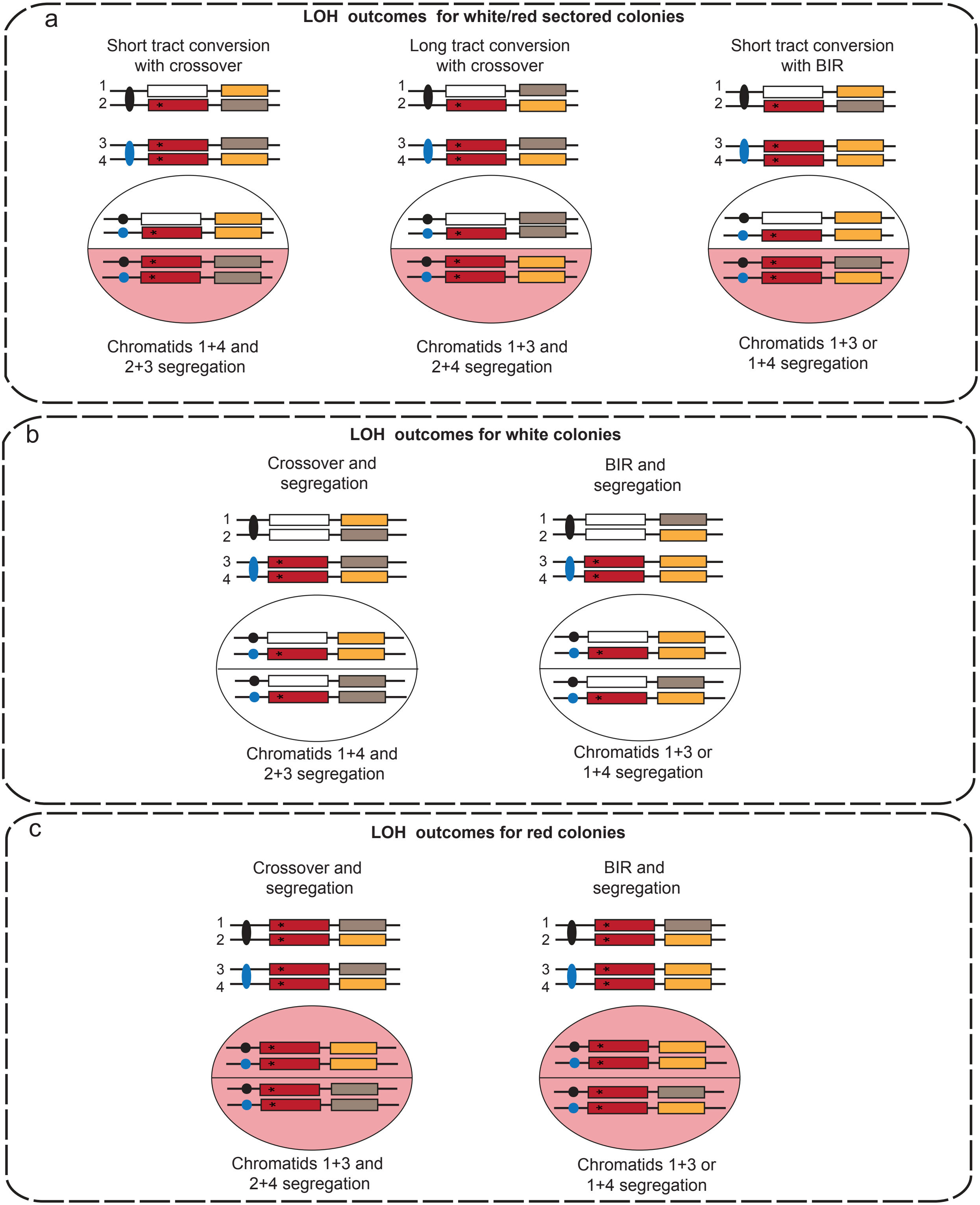

Supplementary Fig. 1: Scheme of the genetic system to test recombination events in LSY2205-11C/LSY2543 background.

Loss of heterozygosity (LOH) outcomes in terms of CO and BIR are shown for white/red colonies (a), white colonies (b) and red colonies (c) with segregation of chromatids.

Ferrari et al SUP. Fig. 2

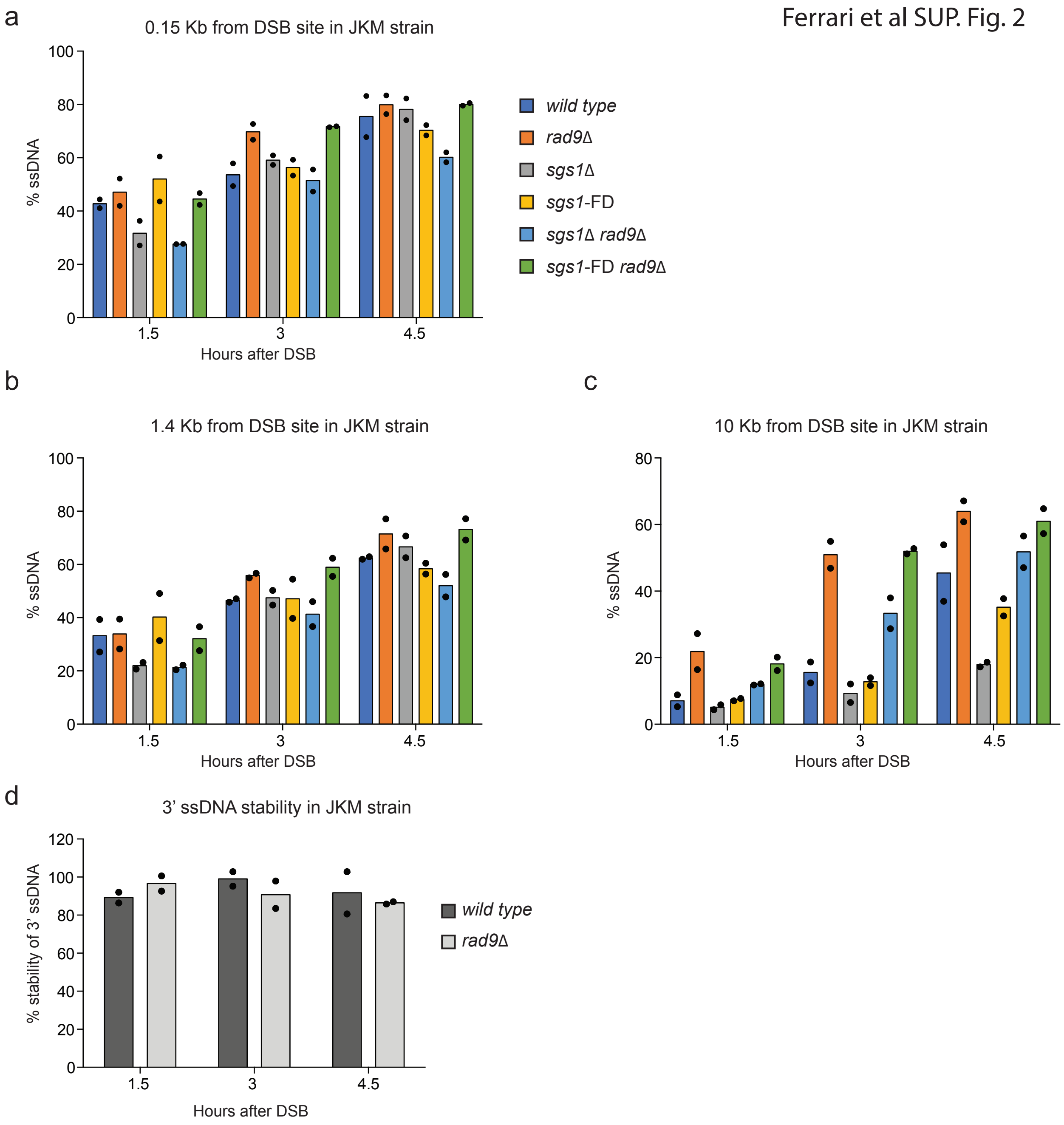

Supplementary Fig. 2: Deletion of *RAD9* and *sgs1*-FD mutation do not alter DSB resection closed to the DSB in JKM139 background.

DSB resection analysis at 0.15 kb (a), 1.4 kb (b) and 10 kb (c) from the DSB on chromosome III in JKM139 derivative strains at the indicated times after HO induction, n=2 biologically independent experiments. The indicated strains were blocked in G2/M with nocodazole.

(d) Percent stability of the 3′ end ssDNA filament at the indicated time points in JKM139 derivative strains relative to unprocessed *KCC4* locus. Samples derived by the same experiments as (a). All the graphical bars shown represent the mean between the two experiments. Source data are provided as a Source Data file.

a

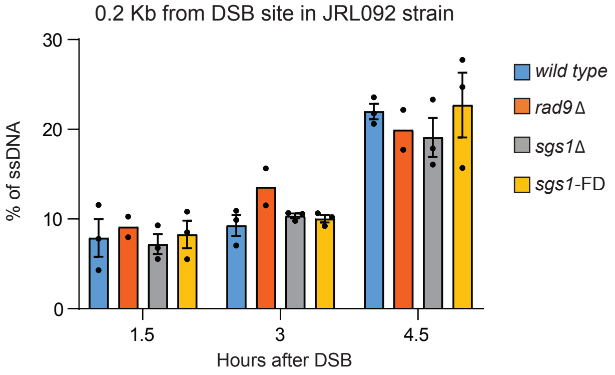
0.2 Kb from DSB site in JRL092 strain
% of ssDNA
30
20
10
0
1.5
3
4.5
Hours after DSB
wild type
rad9Δ
sgs1Δ
sgs1-FD

b

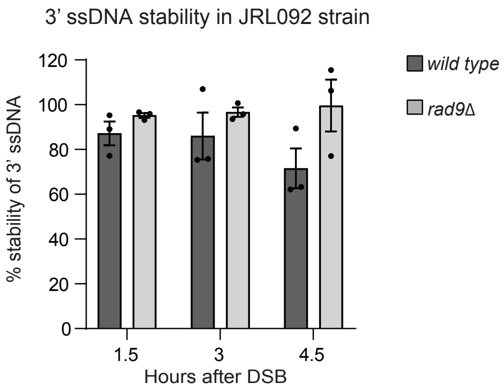
3' ssDNA stability in JRL092 strain
% stability of 3' ssDNA
120
100
80
60
40
20
0
1.5
3
4.5
Hours after DSB
wild type
rad9Δ

Supplementary Fig. 3: Deletion of *RAD9* and *sgs1*-FD mutations do not alter DSB resection closed to the DSB in JRL092 background.

DSB resection analysis at 0.2 kb (a) from the DSB on chromosome V in JRL092 derivative strains at the indicated times after HO induction (n=3, biologically independent samples for wild type, *sgs1*Δ and *sgs1*-FD; n=2 for *rad9*Δ). The indicated strains were blocked in G2/M with nocodazole.

(b) Percent stability of the 3′ end ssDNA filament at the indicated time points in JRL092 derivative strains relative to unprocessed *KCC4* locus. Samples derived by the same experiments as (a).

All the data in the figure are presented as mean ± SEM wherever n=3 or more. Source data are provided as a Source Data file.

Ferrari et al SUP. Fig. 4

a

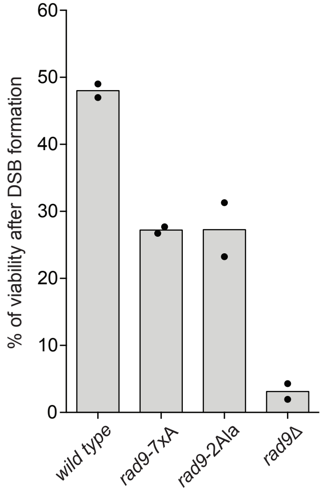

Supplementary Fig. 4: *rad9*-2Ala and *rad9*-7xA alleles partially rescue BIR of *rad9*Δ cells.

BIR efficiency measured by cell viability in the indicated JRL092 strains (n=2, biologically independent experiments). All the data in the figure are presented as mean. Source data are provided as a Source Data file.

a

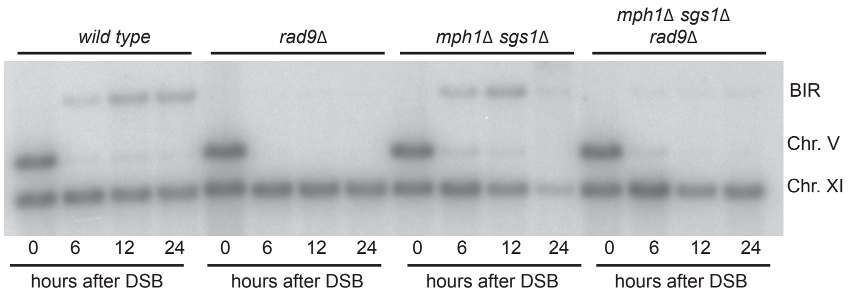
wild type
rad9Δ
mph1Δ sgs1Δ
mph1Δ sgs1Δ rad9Δ
BIR
Chr. V
Chr. XI
0 6 12 24
hours after DSB

b

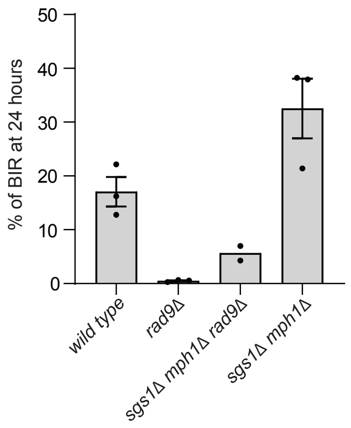
% of BIR at 24 hours
50
40
30
20
10
0
wild type
rad9Δ
sgs1Δ mph1Δ rad9Δ
sgs1Δ mph1Δ

c

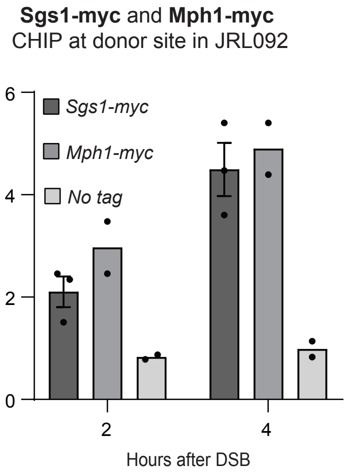
Sgs1-myc and **Mph1-myc**
CHIP at donor site in JRL092
Sgs1-myc
Mph1-myc
No tag
6
4
2
0
2
4
Hours after DSB

Supplementary Fig. 5: Deletion of *SGS1* and *MPH1* partially rescue BIR deficiency of *rad9*Δ cells.

(a) Southern blot of *Ava*I-digested DNA to monitor DSB repair through BIR in the indicated JRL092 derivative strains.

(b) Densitometric analysis of the BIR band as in (a) (n=3 biologically independent experiments for wild type, *rad9*Δ*, sgs1*Δ *mph1*Δ strains; n=2 for *mph1*Δ *sgs1*Δ *rad9*Δ strain).

(c) Mph1 and Sgs1 binding at the donor site on chromosome XI in the indicated JRL092 derivative strains blocked in G2/M with nocodazole, (n=2, biologically independent experiments for the No tag and Mph1-Myc strains; n=3 for the Sgs1-Myc strain). The ‘no-tag’ control strain was added to test the specificity of the 9E10 antibodies in this experimental setting. All data were normalized also to the 0 h time point.

All the data in the figure are presented as mean. SEM is shown wherever n=3. Source data are provided as a Source Data file.
