# Supplementary material for: Rad9/53BP1 promotes DNA repair via crossover recombination by limiting the Sgs1 and Mph1 helicases
Source: Nat Commun. 2020 Jun 23;11:3181. doi: 10.1038/s41467-020-16997-w (PMC7311424; doi:10.1038/s41467-020-16997-w)
Supplement: Supplementary file 3 — Reporting Summary [file 41467_2020_16997_MOESM3_ESM.pdf]

nature research

Corresponding author(s): Achille Pellicioli

Last updated by author(s): May 24, 2020

Reporting Summary

Nature Research wishes to improve the reproducibility of the work that we publish. This form provides structure for consistency and transparency in reporting. For further information on Nature Research policies, see Authors & Referees and the Editorial Policy Checklist.

Statistics

For all statistical analyses, confirm that the following items are present in the figure legend, table legend, main text, or Methods section.

n/a	Confirmed	
☐	☒	The exact sample size (*n*) for each experimental group/condition, given as a discrete number and unit of measurement
☒	☐	A statement on whether measurements were taken from distinct samples or whether the same sample was measured repeatedly
☐	☒	The statistical test(s) used AND whether they are one- or two-sided<br>*Only common tests should be described solely by name; describe more complex techniques in the Methods section.*
☒	☐	A description of all covariates tested
☒	☐	A description of any assumptions or corrections, such as tests of normality and adjustment for multiple comparisons
☐	☒	A full description of the statistical parameters including central tendency (e.g. means) or other basic estimates (e.g. regression coefficient) AND variation (e.g. standard deviation) or associated estimates of uncertainty (e.g. confidence intervals)
☐	☒	For null hypothesis testing, the test statistic (e.g. *F*, *t*, *r*) with confidence intervals, effect sizes, degrees of freedom and *P* value noted<br>*Give P values as exact values whenever suitable.*
☒	☐	For Bayesian analysis, information on the choice of priors and Markov chain Monte Carlo settings
☒	☐	For hierarchical and complex designs, identification of the appropriate level for tests and full reporting of outcomes
☒	☐	Estimates of effect sizes (e.g. Cohen's *d*, Pearson's *r*), indicating how they were calculated

Our web collection on statistics for biologists contains articles on many of the points above.

Software and code

Policy information about availability of computer code

Data collection	no software was used for data collection.
Data analysis	Biorad CFX Manager v. 3.1, Biorad Quantasoft v. 1.7.4.0917, Biorad Image Lab v. 5.2.1, ImageJ 1.44o, GraphPad Prism 8.4.2, Microsoft Excel Professional Plus 2013.

For manuscripts utilizing custom algorithms or software that are central to the research but not yet described in published literature, software must be made available to editors/reviewers. We strongly encourage code deposition in a community repository (e.g. GitHub). See the Nature Research guidelines for submitting code & software for further information.

Data

Policy information about availability of data

All manuscripts must include a data availability statement. This statement should provide the following information, where applicable:

- Accession codes, unique identifiers, or web links for publicly available datasets
- A list of figures that have associated raw data
- A description of any restrictions on data availability

All data are in the paper and supplementary information. The source data underlying Figs. 2b-e; 3a-c; 4b-e; 5a-h; 6b-d; Supplementary Figs. 2a-d; 3a, b; 4a; 5a-c are provided as a Source Data file. All data is available from the authors upon reasonable request.

Field-specific reporting

Please select the one below that is the best fit for your research. If you are not sure, read the appropriate sections before making your selection.

☒ Life sciences ☐ Behavioural & social sciences ☐ Ecological, evolutionary & environmental sciences

For a reference copy of the document with all sections, see nature.com/documents/nr-reporting-summary-flat.pdf

Life sciences study design

All studies must disclose on these points even when the disclosure is negative.

Sample size	all data is from ensemble experiments from >2x10e7 cells. Sample size has been determined by counting cells directly from the culture with Burker chamber. Based on literature and our experince, the sample size is sufficient since this is the minimun number of cells essential to obtain enough DNA materials to perform experiments where DNA extraction is required or enough cells for plating experiments.
Data exclusions	No data were excluded from the analyses.
Replication	All experiments were repeated at least 2 times (see reproducilibility statement in the methods section) to confirm the reproducibility of the data. When applicable, statistical analysis confirmed reproducibility of the results.
Randomization	Randomization are not relevant in our study as we study DSB repair mechanisms in genomic DNA preparations extracted from cell culture clones rather than in specific cells. Cell clones were isolated randomly by genetic procedures.
Blinding	Blinding allocation is not relevant to our studies and does not include any clinical data.

Reporting for specific materials, systems and methods

We require information from authors about some types of materials, experimental systems and methods used in many studies. Here, indicate whether each material, system or method listed is relevant to your study. If you are not sure if a list item applies to your research, read the appropriate section before selecting a response.

Materials & experimental systems

n/a	Involved in the study	
☐	☒	Antibodies
☒	☐	Eukaryotic cell lines
☒	☐	Palaeontology
☒	☐	Animals and other organisms
☒	☐	Human research participants
☒	☐	Clinical data

Methods

n/a	Involved in the study	
☒	☐	ChIP-seq
☒	☐	Flow cytometry
☒	☐	MRI-based neuroimaging

Antibodies

Antibodies used	PA5-34905 (Anti-yRad51) by Thermo Fischer Scientific; 9E10 (Anti MYC tag), home-made production; 12CA5 (Anti HA tag), home-made production. We did not perform western blotting or any other application based on antibody dilution.
Validation	As for the Rad51 ChIP, we used the previously reported polyclonal anti-Rad51 that were already used for similar application (Graf et al. Cell, 2017, 170 (19:72-85. doi: 10.1016/j.cell.2017.06.006) and validated by Thermo Fischer Scientific.<br>As for the 9E10 antibody that we used for ChIP assays, we did a control test for the specificity for the MYC-tagged Sgs1 and Mph1 variants in Supplementary Fig. 5d.<br>In past, we tested the specificity of 12CA5 antibodies that we used for Rpa1-HA and Rad52-HA ChIP analyses at cut site in JKM139 derivatives (Donnianni et at, PLoS Genet. 2015 Jan 8;11(1):e1004928. doi: 10.1371/journal.pgen.1004928). Based on huge literature and long experience in our lab, they are highly specific for this application.
